# The efficacy and safety of fluvoxamine in patients with COVID-19: A systematic review and meta-analysis from randomized controlled trials

**DOI:** 10.1371/journal.pone.0300512

**Published:** 2024-05-16

**Authors:** Qiufeng Zhou, Guozheng Zhao, Yu Pan, Ying Zhang, Yuehua Ni

**Affiliations:** 1 Department of Neurosurgery, Suzhou Ninth People’s Hospital, Suzhou, Jiangsu Province, China; 2 Department of Emergency, Suzhou Ninth People’s Hospital, Suzhou, Jiangsu Province, China; The Islamia University of Bahawalpur Pakistan, PAKISTAN

## Abstract

**Background:**

Recently, several randomized controlled trials (RCTs) of fluvoxamine have been successfully conducted for the treatment of patients with coronavirus disease 2019 (COVID-19). This systematic review and meta-analysis was to evaluate the efficacy and safety of fluvoxamine in patients with COVID-19.

**Methods:**

MEDLINE, EMBASE, Cochrane Library and clinicaltrials.gov were searched for RCTs which were performed to evaluate fluvoxamine and placebo up to January 31, 2024. Review Manager 5.3 was used to perform meta-analysis. The risk ratio (RR) and mean difference (MD) was analyzed and calculated with a random effect model.

**Results:**

We pooled 4,711 participants from six RCTs (2,382 in the fluvoxamine group and 2,329 in the placebo group). Compared to the placebo group, the fluvoxamine group had a significantly lower rate of clinical deterioration (RR, 0.73; P = 0.004; 95% CI, 0.59 to 0.90; I^2^ = 0%) and hospitalization (RR, 0.76; P = 0.04; 95% CI, 0.59 to 0.99; I^2^ = 0%). In the meantime, compared with the placebo group, fluvoxamine group did not show any higher risk of AEs (P = 0.13 and 0.91, respectively) in safety outcomes analysis. The subgroup analysis showed that fluvoxamine treatment performed more than 200 mg daily appears to be more effective than those performed less than 200 mg daily in reducing clinical deterioration and hospitalization risks, while not exhibiting higher AE and SAE risks than placebo group.

**Conclusion:**

Fluvoxamine for patients with COVID-19, especially those who take 200 mg or more daily, is superior to the placebo group in reducing clinical deterioration and hospitalization, and did not show any higher risk of AEs and SAEs in safety concerns, which might be a promising intervention for COVID-19.

## 1. Introduction

The coronavirus illness 2019 (COVID-19), which was caused by the severe acute respiratory syndrome coronavirus 2 (SARS-CoV-2) infection, has had a considerable detrimental impact on the global economy and presented various health risks. According to the World Health Organization’s (WHO) imperfect statistics, the COVID-19 outbreak has lasted for more than three years and as of February 2024, there have been more than 775 million confirmed cases of the virus, including more than 7 million fatalities [1]. The SARS-CoV-2 virus not only irritates the respiratory system but also the heart, gastrointestinal tract, liver, kidney, and central nervous system, which can ultimately lead to multiorgan failure [2]. Although some direct antiviral drugs and engineered monoclonal antibodies have been approved/authorized by FDA or other organizations, there are challenges in terms of availability, accessibility, management, and affordability in most parts of the world [3], so there is still need for more treatment options that are less expensive and more widely available.

Increasing evidence indicates that cytokine storm syndrome may be present in a subset of people with severe COVID-19 [4]. Therefore, it is essential to modulate cellular immunity and the inflammatory response in the treatment of COVID-19. Aganism of the sigma-1 receptor (S1R) is one potential immune control strategy. As an endoplasmic reticulum chaperone protein, controlling cytokine production is one of the numerous biological functions of the S1R [5]. Fluvoxamine, a well-known, frequently used, and reasonably priced selective serotonin reuptake inhibitor (SSRI) was initially licensed by the US Food and Drug Administration in 1994 to treat obsessive-compulsive disorder, was recently investigated in the context of acute COVID-19 infection [6]. Fluvoxamine may be advantageous in cases of acute COVID-19 infection, according to a few proposed pathways. Firstly, the S1R is known to be activated by fluvoxamine, which reduces the production of pro-inflammatory cytokines [7]. Second, fluvoxamine inhibits the acid sphingomyelinase/ceramide pathway that SARS-CoV-2 depends on for viral entry because it is a functional inhibitor of acid sphingomyelinase activity [8].

A number of meta-analyses for pharmacological interventions of COVID-19 have been published in an effort to gather data from individual trials evaluating different interventions in order to identify successful COVID-19 treatments, fluvoxamine was also no exception. The effects of fluvoxamine were well verified in several large randomized controlled trials (RCTs) in the treatment of COVID-19 patients [9–14], however, the results of previously published meta-analyses of fluvoxamine in the treatment of COVID-19 did not appear to be entirely consistent due to their different inclusion and exclusion criteria (such as including both RCTs and prospective cohort studies). In addition, recently published RCTs have also explored the dosage used of fluvoxamine [14]. Clinicians need sufficient evidence to make the best clinical choice for each patient with COVID-19. Thus, we pooled data from previous RCTs and conducted a systematic review and meta-analysis to investigate the efficacy and safety of fluvoxamine for patients with COVID-19.

## 2. Methods

### 2.1 Study protocol

We created a research protocol in accordance with the Cochrane Collaboration format before the investigation began [15]. The systematic review’s protocol was registered on INPLASY (INPLASY 202330078), and it is fully accessible there (10.37766/inplasy2023.3.0078).

### 2.2 Eligibility criteria

We established the following inclusion standards: (1) study type: RCT; (2) language restriction: English only; (3) participants: patients over the age of 18 who have been diagnosed with SARS-CoV-2 infection; (4) intervention: fluvoxamine and a matched placebo; (5) outcomes: efficacy outcomes including the number of patients who experience clinical deterioration, the number of patients who require hospitalization, the number of patients who require mechanical breathing, and the length of time before clinical deterioration. Clinical deterioration was a composite outcome that included hypoxemia, a trip to the emergency department, an urgent care visit, a hospital stay, or death. Regardless of whether the patient receives medical care, clinical deterioration can be defined as a further decline in the patient’s state. Adverse events (AEs) and serious adverse events (SAEs) are included in our safety outcomes. As an exploratory outcome, mortality was also defined. All of the aforementioned outcomes were not required to be provided by the included RCTs.

We established the following exclusion standards: (1) research design: retrospective studies, cohort studies, case reviews, and case reports; (2) control: active control (i.e., comparison of an experimental treatment with a known, successful treatment rather than a placebo).

### 2.3 Search strategy

Two independent investigators (QFZ and GZZ) systematically searched MEDLINE, EMBASE, Cochrane Library and the ClinicalTrials.gov to identify relevant studies published until January 31, 2024. The following search strategy was employed: (fluvoxamine [Title/Abstract]) AND (COVID-19 [Title/Abstract]) for MEDLINE; `fluvoxamine’/exp AND `COVID-19’/exp for EMBASE; "fluvoxamine" in Title Abstract Keyword AND "COVID-19" in Title Abstract Keyword for Cochrane Library; “fluvoxamine | COVID-19” for ClinicalTrials.gov. The detailed search strategy can be found in the electronic supplementary material (**S1 Table**). Additionally, the reference lists of RCTs, relevant systematic reviews and meta-analyses were also screened independently and manually to ensure a more comprehensive search.

### 2.4 Study selection and data collection

Based on the previously mentioned eligibility criteria, two authors (QFZ and GZZ) independently analyzed all records found in the electronic database as well as the references of relevant RCTs, systematic reviews, and meta-analyses. Duplicate research and articles with only abstracts were eliminated. The two writers’ disagreements were settled by conversation or, if necessary, by a third author (YP), who was not involved in the data collection process. After selection and evaluation, all information from the included RCTs was extracted in the following ways: fundamental details and outcome events for each RCT were included in **Table 1**; inclusion and exclusion criteria, study design, and all efficacy and safety outcomes were shown in the online supplementary materials (**S2 Table**).

**Table 1:** Characteristics of the included studies and outcome events.

Study	Countries	Centers	Treatment group, (No. of participants)	Male (%)	Mean age ±SD (year)	Study period	Duration of COVID-19 symptoms	Dosage of Fluvoxamine	Outcome Events
Lenze et al 2020 (STOP COVID 1)	USA	1	Fluvoxamine (80) vs. Placebo (72)	Fluvoxamine: 30 Placebo: 26	Fluvoxamine: 46.4±14.3 Placebo: 45±11.8	15 days	Fluvoxamine: 4±1.4 Placebo: 4±1.4	100 mg three times daily	a, b, c, e, f, g
Reis et al 2021 (TOGETHER)	Brazil	11	Fluvoxamine (741) vs. Placebo (756)	Fluvoxamine: 45 Placebo: 40	Fluvoxamine: 48.2±12.6 Placebo: 47.6±13.4	28 days	NR	100 mg twice daily	a, b, c, d, e, f, g
Seo et al 2022	Korea	1	Fluvoxamine (26) vs. Placebo (26)	Fluvoxamine: 69 Placebo: 50	Fluvoxamine: 52.7±12.8 Placebo: 50.9±13.6	10 days	NR	100 mg twice daily	a, d, e, g
McCarthy et al 2023 (ACTIV-6 Low)	USA	91	Fluvoxamine (674) vs. Placebo (614)	Fluvoxamine: 42 Placebo: 43	Fluvoxamine: 46.9±14.7 Placebo: 48.4±14.1	28 days	Fluvoxamine: 5.4±2.2 Placebo: 5.4±2.2	50 mg twice daily	a, b, d, e, f, g
Reiersen et al 2023 (STOP COVID 2)	USA and Canada	5	Fluvoxamine (272) vs. Placebo (275)	Fluvoxamine: 38 Placebo: 38	Fluvoxamine: 48±10.2 Placebo: 48±9.8	15 days	Fluvoxamine: 5±1.4 Placebo: 4.8±1.5	100 mg twice daily	a, e, f, g
Stewart et al 2023 (ACTIV-6 High)	USA	103	Fluvoxamine (589) vs. Placebo (586)	Fluvoxamine: 35 Placebo: 34	Fluvoxamine: 50.0±16.3 Placebo: 50.4±14.1	28 days	Fluvoxamine: 3.4±2.2 Placebo: 3.6±2.2	100 mg twice daily	a, b, e, f, g

COVID-19: coronavirus disease 2019; a: the number of patients with clinical deterioration; b: the number of patients with hospitalisation; c: the number of patients with mechanical ventilation; d: time to clinical deterioration; e: adverse events; f: serious adverse events; g: death; NR: not reported.

### 2.5 Risk of bias

Review Manager 5.3 was used to assess the risk of bias plot. The risk of bias for RCTs was evaluated using the universal Cochrane Collaboration criteria [16], which took into account selection bias, performance bias, detection bias, attrition bias, reporting bias, and other potential biases. Each bias criterion was assigned one of three levels: "low," "high," or "unclear.". QFZ and GZZ conducted the assessment independently. When there were disagreements, a third author (YP) was consulted.

### 2.6 Summary measures and synthesis of results

Direct evidence was meta-analyzed in pairs using Review Manager 5.3 software. For the dichotomous outcomes, the relative risk (RR) with a 95% confidence interval (95% CI) was examined and determined. The continuous outcome "the time to clinical deterioration" was the only one for which the mean difference (MD) was employed. The I^2^ statistic was used to measure heterogeneity, and the results were as follows: I^2^ indicates "low heterogeneity" when it is below 30%, "moderate heterogeneity" when it is between 30% and 50%, and "substantial heterogeneity" when it is above 50%. Additionally, sensitivity analysis was done to investigate the stability of the combined data. We also implemented subgroup analyses to detect the effect of different doses of fluvoxamine at baseline on efficacy and safety outcomes. Random effect model and two-tailed tests were used for all of the analyses, and a P value of 0.05 or lower was regarded as statistically significant.

## 3. Results

### 3.1 Search results and study characteristics

**Fig 1:**
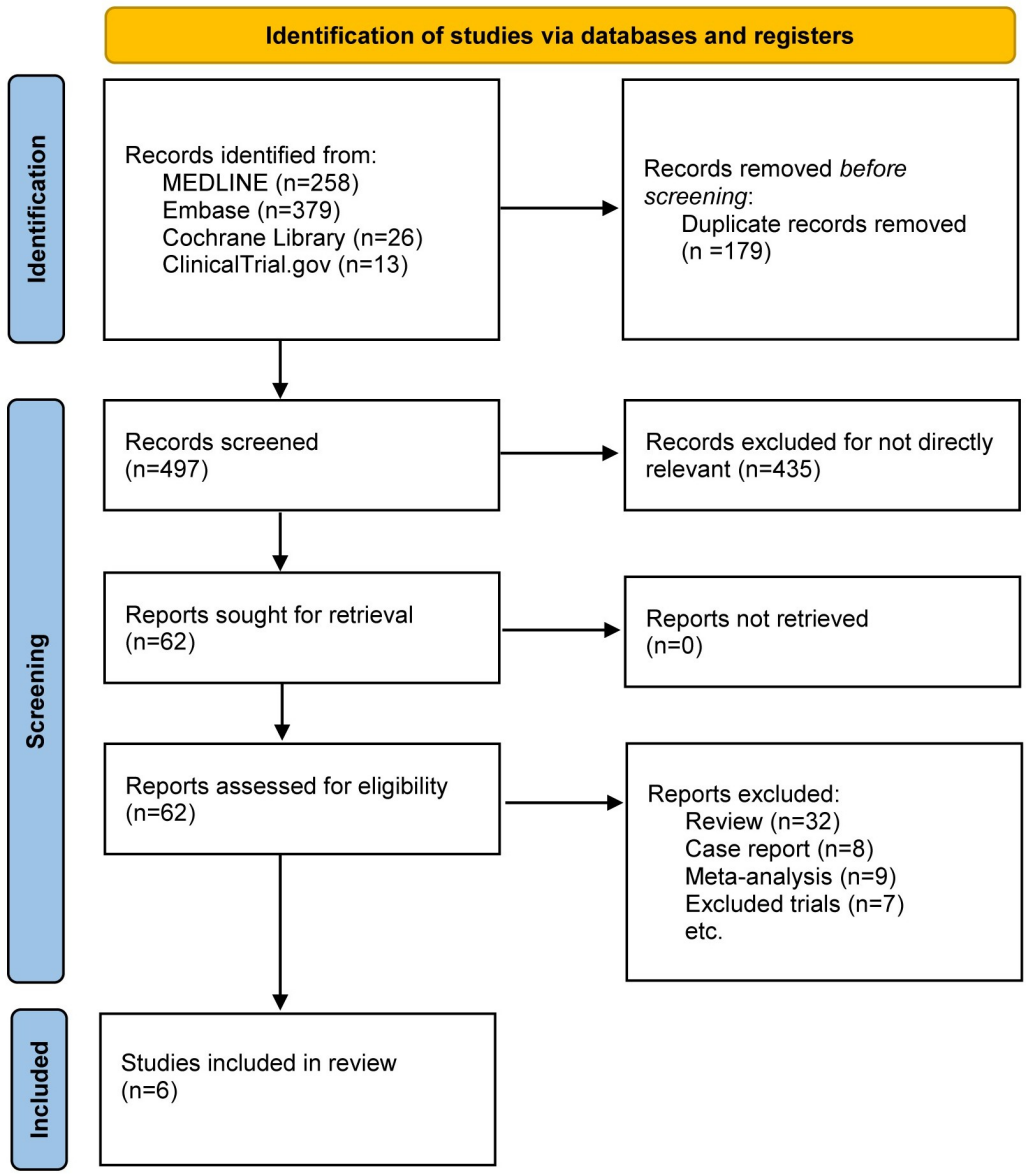
The study search, selection, and inclusion process.

Overall, there were 676 titles and abstracts from the Cochrane Library, MEDLINE, EMBASE, and Clinicaltrials.gov databases. After a brief review, 614 items were excluded due to duplication or irrelevance, and 62 complete articles were assessed for eligibility. Eight case reports, nine meta-analyses, 32 reviews, and seven non-randomized clinical trials were among the 62 papers that were disqualified because they did not meet the requirements for their publication type. In the end, six RCTs with 4,711 participants (2,382 in the fluvoxamine group and 2,329 in the placebo group, **Fig 1**) were selected for qualitative synthesis. **Table 1** is a summary of the key traits of the six studies that were included.

### 3.2 Efficacy outcome

**Fig 2:**
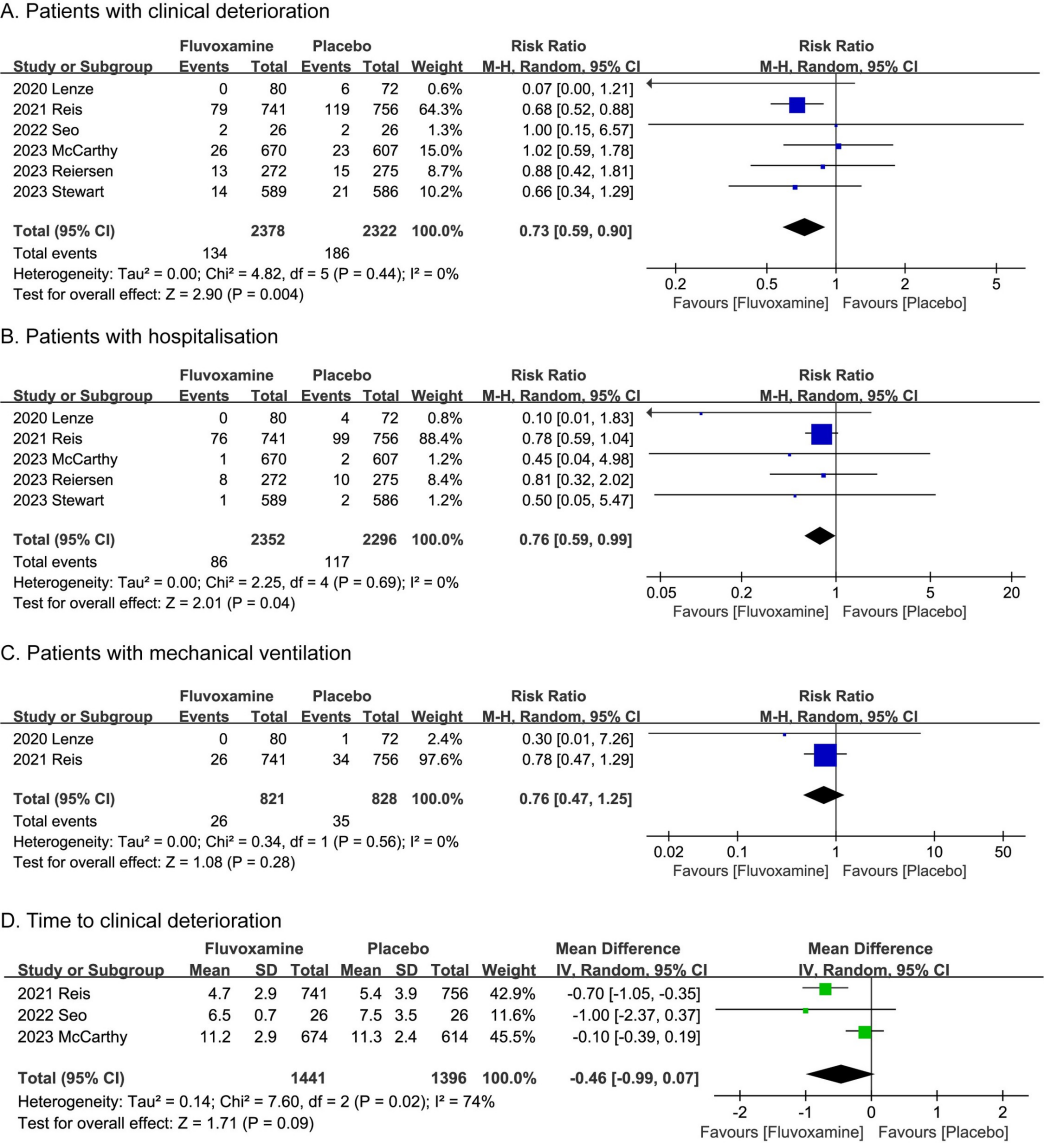
Meta-analysis results of efficacy outcomes. **A.** The number of patients with clinical deterioration. **B.** The number of patients with hospitalization. **C.** The number of patients with mechanical ventilation. **D.** The time to clinical deterioration.

In this meta-analysis, efficacy outcomes including the number of patients with clinical deterioration, the number of patients with hospitalization, the number of patients with mechanical ventilation and the time to clinical deterioration. The fluvoxamine group showed a significantly lower rate of clinical deterioration than the placebo group (RR, 0.73; P = 0.004; 95% CI, 0.59 to 0.90; I^2^ = 0%; **Fig 2A**). At the same time, patients in fluvoxamine group also had significantly lower probability to be hospitalized than those in the placebo group (RR, 0.76; P = 0.04; 95% CI, 0.59 to 0.99; I^2^ = 0%; **Fig 2B**). However, for the collected data, patients in fluvoxamine group did not show significant differences in the number of patients with mechanical ventilation and the time to clinical deterioration when compared with the patients in placebo group (P = 0.28 and 0.09, respectively, **Fig 2C and 2D**).

### 3.3 Safety and exploratory outcome

**Fig 3:**
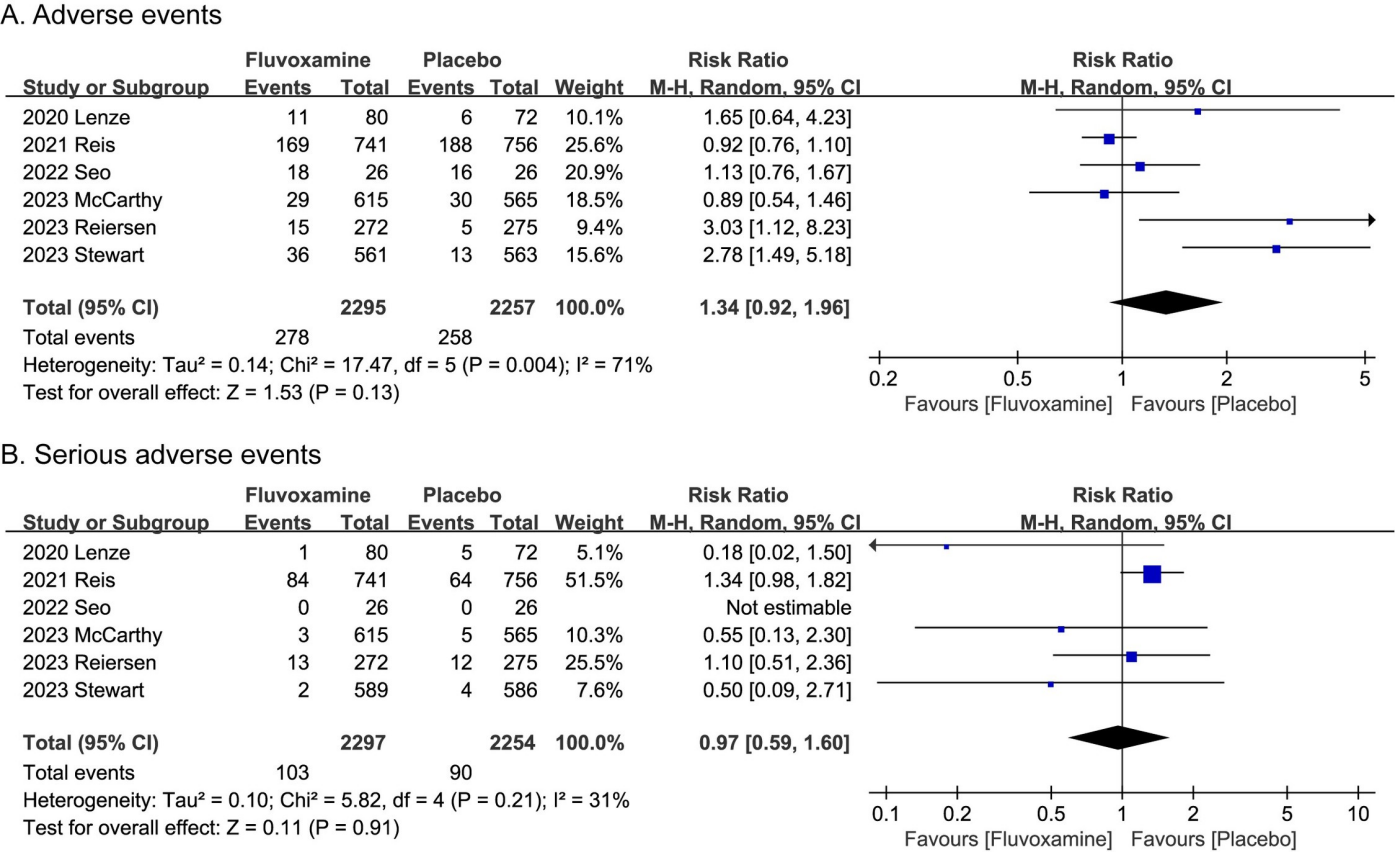
Meta-analysis results of safety outcomes. **A.** Adverse events. **B.** Serious adverse events.

**Fig 4:**
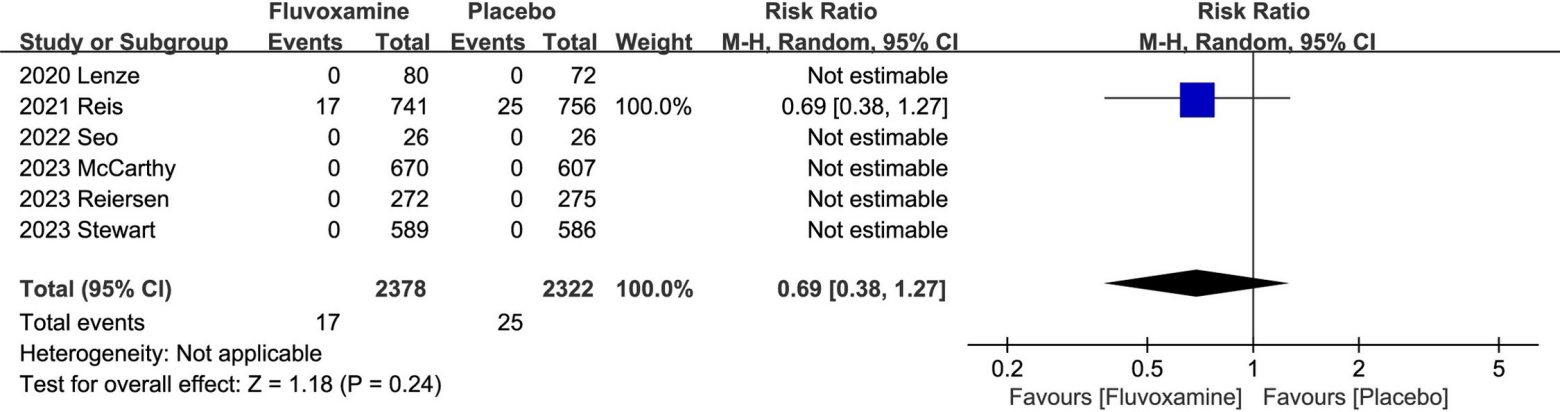
Meta-analysis results of exploratory outcome: Mortality.

The safety outcomes were assessed by AEs and SAEs. We combined the data collected from the six trials and found that fluvoxamine did not show a significant high risk of the AEs (RR, 1.34; P = 0.13; 95% CI, 0.92 to 1.96; I^2^ = 71%; **Fig 3A**) and the SAEs (RR, 0.97; P = 0.91; 95% CI, 0.59 to 1.60; I^2^ = 31%; **Fig 3B**). Sensitivity analysis was also performed for AEs (I^2^ = 71%), and it demonstrated that if we excluded the ACTIV-6 High trial, the overall heterogeneity would have been less than 50%, while the difference between two groups remains not significant (**S1 Fig**). In addition, we also classified mortality as an exploratory outcome to explore whether fluvoxamine can reduce the number of deaths caused by COVID-19 infection. Of the six studies included, only Reis et al. reported that there were deaths during the whole study period, including 17 deaths in group fluvoxamine and 25 deaths in group placebo (RR, 0.69; P = 0.24; 95% CI, 0.38 to 1.27; **Fig 4**). The other five studies both reported no deaths during the whole study period.

### 3.4 Subgroup analyses

To assess the influence of different doses of fluvoxamine (low dose: < 100 mg twice daily; high dose: ≥ 100 mg twice daily), we implemented subgroup analyses at baseline. The results indicated that, compared to the placebo group, the high dose fluvoxamine group showed a significantly lower rate of clinical deterioration and hospitalization (P = 0.001 and 0.05, respectively). However, patients in low dose fluvoxamine group did not show lower probability to be deteriorated or hospitalized than those in the placebo group (P = 0.93 and 0.52, respectively). In the subgroup analysis of safety outcomes, we found that neither the high dose fluvoxamine group nor the low dose fluvoxamine group showed a significant high risk of AEs and SAEs than the placebo group (all P > 0.05). The detailed results of the subgroup analyses are shown in **Table 2** and **S2–S5 Figs**.

**Table 2:** Subgroup analysis of efficacy and safety outcomes.

	Low dose Fluvoxamine		High dose Fluvoxamine	
	RR [95% CI]	*p* value	RR [95% CI]	*p* value
**Efficacy outcomes**				
Number of patients with clinical deterioration	1.02 [0.59, 1.78]	0.93	0.69 [0.55, 0.87]	0.001
Number of patients with hospitalisation	0.45 [0.04, 4.98]	0.52	0.77 [0.59, 1.00]	0.05
**Safety outcomes**				
AEs	0.89 [0.54, 1.46]	0.64	1.52 [0.95, 2.43]	0.08
SAEs	0.55 [0.13, 2.30]	0.41	1.03 [0.60, 1.76]	0.92

RR: Relative Risk; CI: Confidence Interval; AEs: Adverse Events; SAEs: Serious Adverse Events.

### 3.5 Risk of bias in included studies

**Fig 5:**
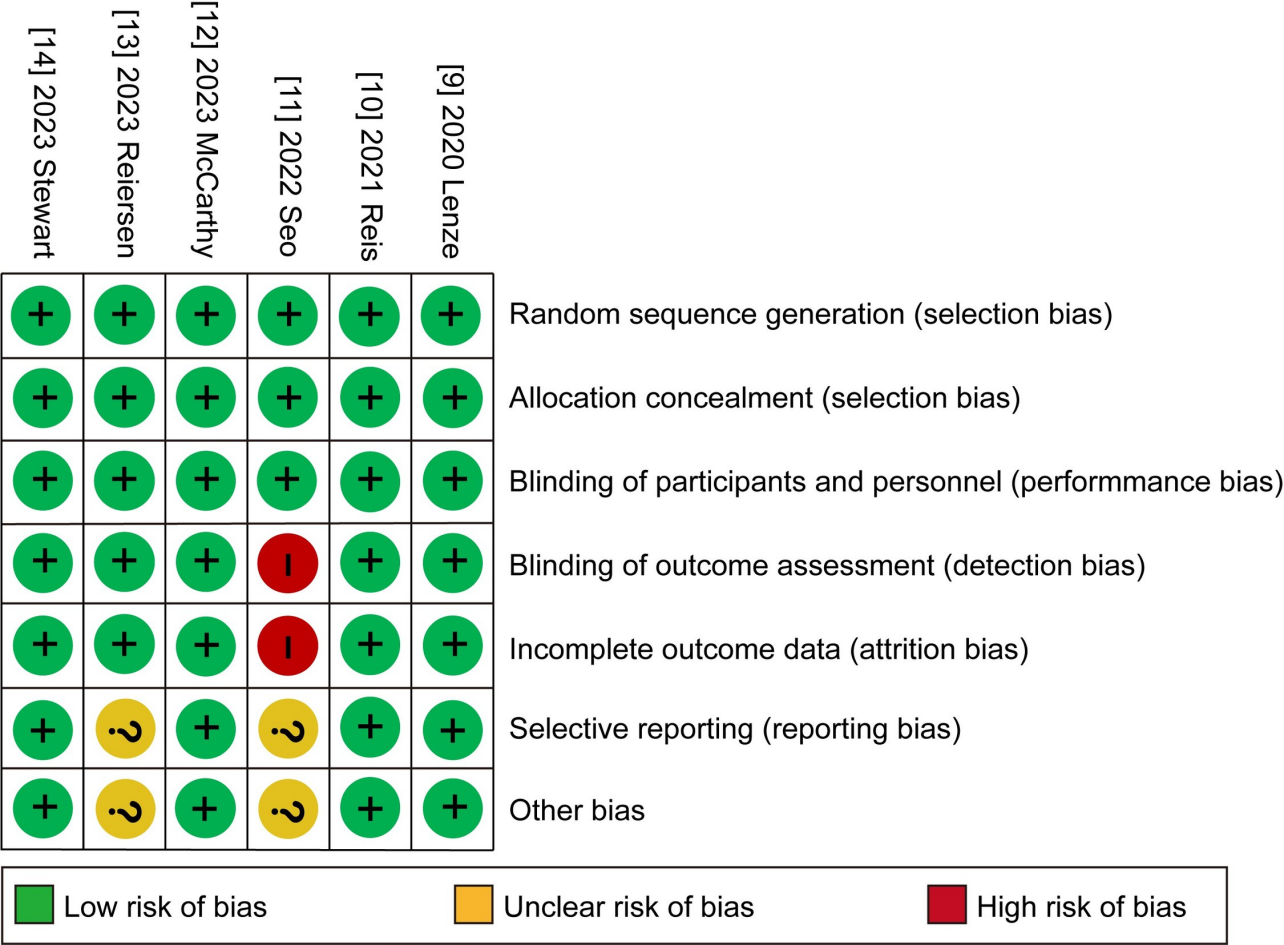
Risk of bias: A summary table for each risk of bias item for each study.

Full details of the risk bias for all enrolled studies were showed in **Fig 5**. All clinical trials showed low risk of bias both in random sequence generation and allocation concealment. For the blinding of participants and personnel, the risk of bias was low in all trials. For the blinding of outcome assessment, the risk of bias was high in one study. For the incomplete outcome data, the risk of bias was also high in the same trial. For selective reporting, the risk of bias was unclear in two study. Apart from these items, two unclear risk of bias was also observed in the same RCTs [11, 13].

## 4. Discussion

In general, the present study is based on six RCTs, which included 4,711 COVID-19 patients randomly assigned to fluvoxamine or placebo group. The results of our meta-analysis presented that for efficacy outcomes analysis, adult patients with COVID-19 in fluvoxamine group, especially those who take 200 mg or more daily, presented significantly lower rate in clinical deterioration and hospitalization compared with those in placebo group. In the meantime, compared with the placebo group, fluvoxamine group did not show any higher risk of AEs and SAEs in safety outcomes analysis, whether in the high-dose or low-dose groups. Given that only one study has reported deaths, we have not yet figured out whether fluvoxamine has an effect on COVID-19 induced deaths.

Several systematic reviews and meta-analyses have also reported on this issue; however, their conclusions are not totally consistent. The meta-analysis of Bhuta et al. [17], which looked at two RCTs and one prospective cohort trial for mechanical ventilation rate and all-cause mortality, reached a similar conclusion as our meta-analysis, which is that the available data does not indicate a significant impact of fluvoxamine on the rates of mechanical ventilation and mortality of patients with COVID-19 infection. Nevertheless, our findings regarding the rates of hospitalization were not supported by Bhuta et al. [17]. They found that in the patients who received fluvoxamine compared with placebo, there was no significant difference in rates of hospitalization. Similar results were also obtained from the meta-analysis conducted by Cheema et al. [18]. However, following the publication of the TOGETHER study results [10], Marcec et al. reanalyzed the hospitalization outcome using the information provided by Cheema while also included hospitalization data from the TOGETHER trial [19]. They found that fluvoxamine does, in fact, statistically significantly lower the probability of hospitalization in COVID-19 outpatients, which was in line with our findings.

It is worth noting that because different studies were included in the present meta-analysis and these two meta-analyses, the findings are only loosely comparable in terms of substance. Both Bhuta et al. and Cheema et al. included the prospective cohort study, while only RCT was included in this meta-analysis. To provide evidence-based recommendations, it is very convincing to analyze only RCTs because of their higher level of evidence. A meta-analysis published in JAMA Network Open that consisted of three RCTs showed a significant reduction in hospitalization rate in patients with COVID-19 receiving fluvoxamine compared to placebo [20]. They included a total of 2,196 subjects, while this article included 3,536 subjects, further validating their results regarding the significant reduction in hospitalization risk of COVID-19 patients with fluvoxamine. Moreover, we further found that fluvoxamine can also significantly reduce the risk of disease deterioration in patients with COVID-19, while a recently published meta-analysis suggested that this effect was still uncertain [21]. It is worth mentioning that their study included the COVID-OUT study, while this study was excluded from the present meta-analysis. The COVID-OUT trial, a double-blind, randomized, placebo-controlled study involving 1431 outpatients with COVID-19, reported that fluvoxamine 50 mg twice daily for 14 days was ineffective in preventing hypoxemia, visits to the emergency room, hospitalization, or deaths linked to COVID-19 [22]. However, due to the design of the trial, there is a positive control in the control group of fluvoxamine that may have a certain therapeutic effect on COVID-19, which will bring some errors, thus, this study was excluded according to our exclusion criteria mentioned above.

All published meta-analyses cited earlier in this article did not undertake dose subgroup analyses, possibly due to the limits of the research they included, thus they cannot give clinicians the evidence about the dosage used of fluvoxamine. The existing RCTs contained multiple fluvoxamine doses, including 50 mg twice daily, 100 mg twice daily and 100 mg three times daily. The TOGETHER trial found that 100 mg fluvoxamine twice daily performed significantly better than placebo, with a 32% reduction in the key composite end point of hospitalization or extended care in an emergency situation [10]. In contrast, the COVID-OUT and ACTIV-6 trials, which studied fluvoxamine 50mg twice daily, found no benefit [12, 22]. As the dose of the drug increases, the effectiveness tends to increase and the safety problem will gradually become prominent, as well as ACTIV-6 has also completed a high-dose fluvoxamine arm recently. Therefore, we conducted a subgroup analysis on the dosage of fluvoxamine. According to our subgroup analysis, fluvoxamine treatment performed more than 200 mg daily appears to be more effective than those performed less than 200 mg daily in reducing clinical deterioration and hospitalization risks, while not exhibiting high AE and SAE risks.

Several limitations of the present meta-analysis should not be ignored. Firstly, the analysis was performed based on limited data. Despite an extensive search, only six published RCTs were pooled to test the effects of fluvoxamine. Secondly, in the analysis of the time to clinical deterioration and AEs, high level of heterogeneity was found. Further sensitivity analysis found that this high heterogeneity was mainly caused by the ACTIV-6 study [12, 14]. The ACTIV-6 trial primarily enrolled the patients with mild to moderate COVID-19, while these patients may have a relatively long time of disease progression. Thirdly, due to the unavailability of individual data, we were unable to conduct subgroup analysis on whether or not vaccines have been administered. As mentioned above, the TOGETHER trial demonstrated that 100 mg fluvoxamine twice daily could bring a 32% reduction in key composite endpoint, while subsequent trials conducted in a majority-vaccinated population showed a 50% reduction in the same composite endpoint [23]. Therefore, the protective effect of vaccines on the deterioration of the disease cannot be ignored.

## 5. Conclusion

In conclusion, the present study indicated that fluvoxamine for patients with COVID-19, especially those who take 200 mg or more daily, is superior to the placebo group in reducing clinical deterioration and hospitalization, and did not show any higher risk of AEs and SAEs in safety concerns. From a comprehensive point of view, fluvoxamine is a promising therapy for patients with COVID-19. More large-scale, high-quality researches are needed to identify further strategies for COVID-19 treatment.

## Supporting information

S1 Fig Sensitivity analysis of the data with heterogeneity greater than 50%: AEs without ACTIV-6 high trial. (TIF)

S2 Fig Subgroup analysis of efficacy: Number of patients with clinical deterioration. (TIF)

S3 Fig Subgroup analysis of efficacy: Number of patients with hospitalisation. (TIF)

S4 Fig Subgroup analysis of safety: AEs. (TIF)

S5 Fig Subgroup analysis of safety: SAEs. (TIF)

S1 Table Detailed search strategy. (DOCX)

S2 Table Inclusion, exclusion criteria, study design and outcome assessments of the included studies. (DOCX)

S1 Checklist PRISMA checklist. (DOCX)
