# Supplementary figures and images for: The efficacy and safety of fluvoxamine in patients with COVID-19: A systematic review and meta-analysis from randomized controlled trials
Source: PLoS One. 2024 May 16;19(5):e0300512. doi: 10.1371/journal.pone.0300512 (PMC11098472; doi:10.1371/journal.pone.0300512)

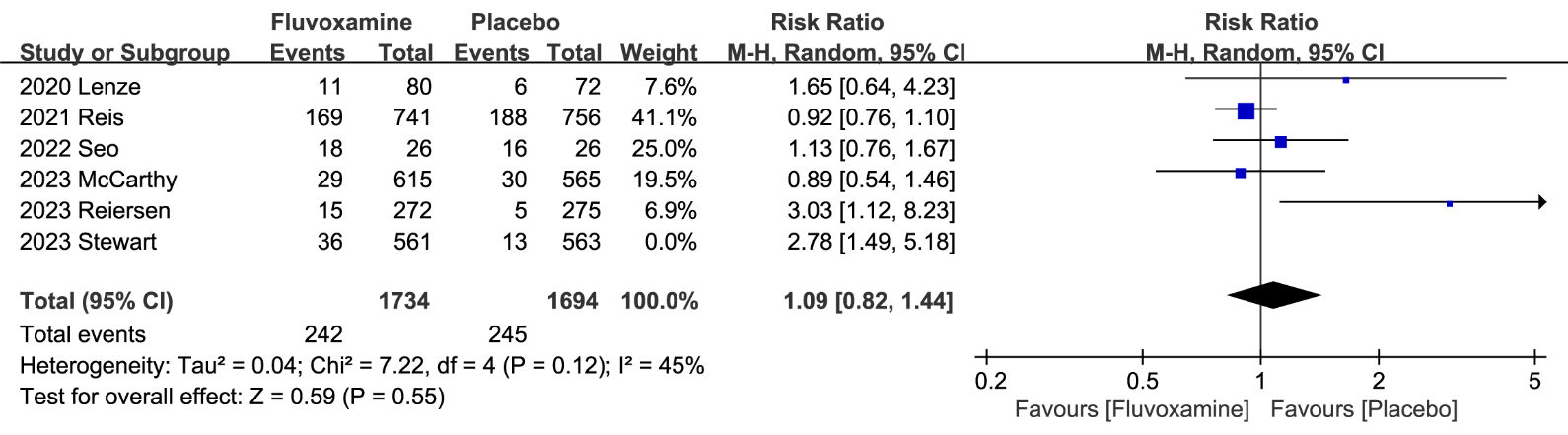

Supplement: S1 Fig — (TIF) [file pone.0300512.s001.tif]

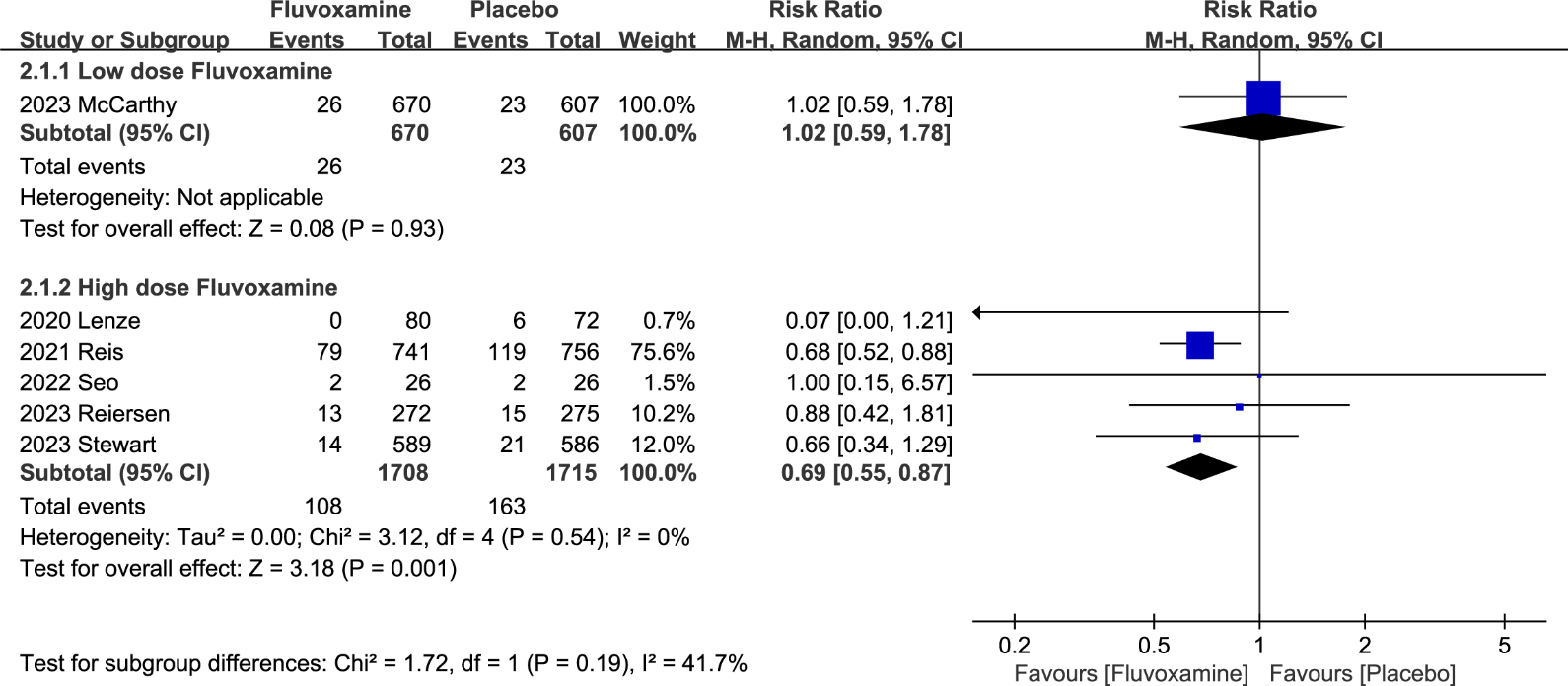

Supplement: S2 Fig — (TIF) [file pone.0300512.s002.tif]

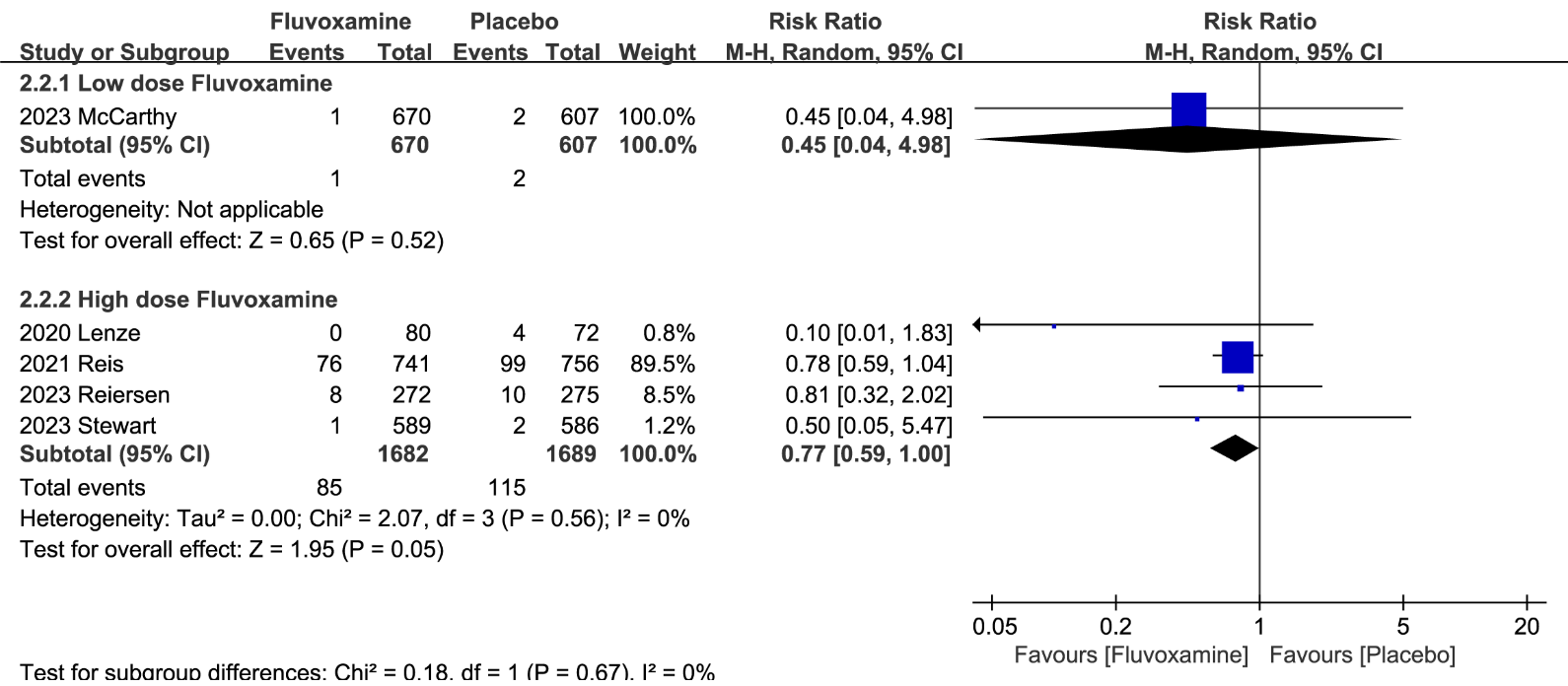

Supplement: S3 Fig — (TIF) [file pone.0300512.s003.tif]

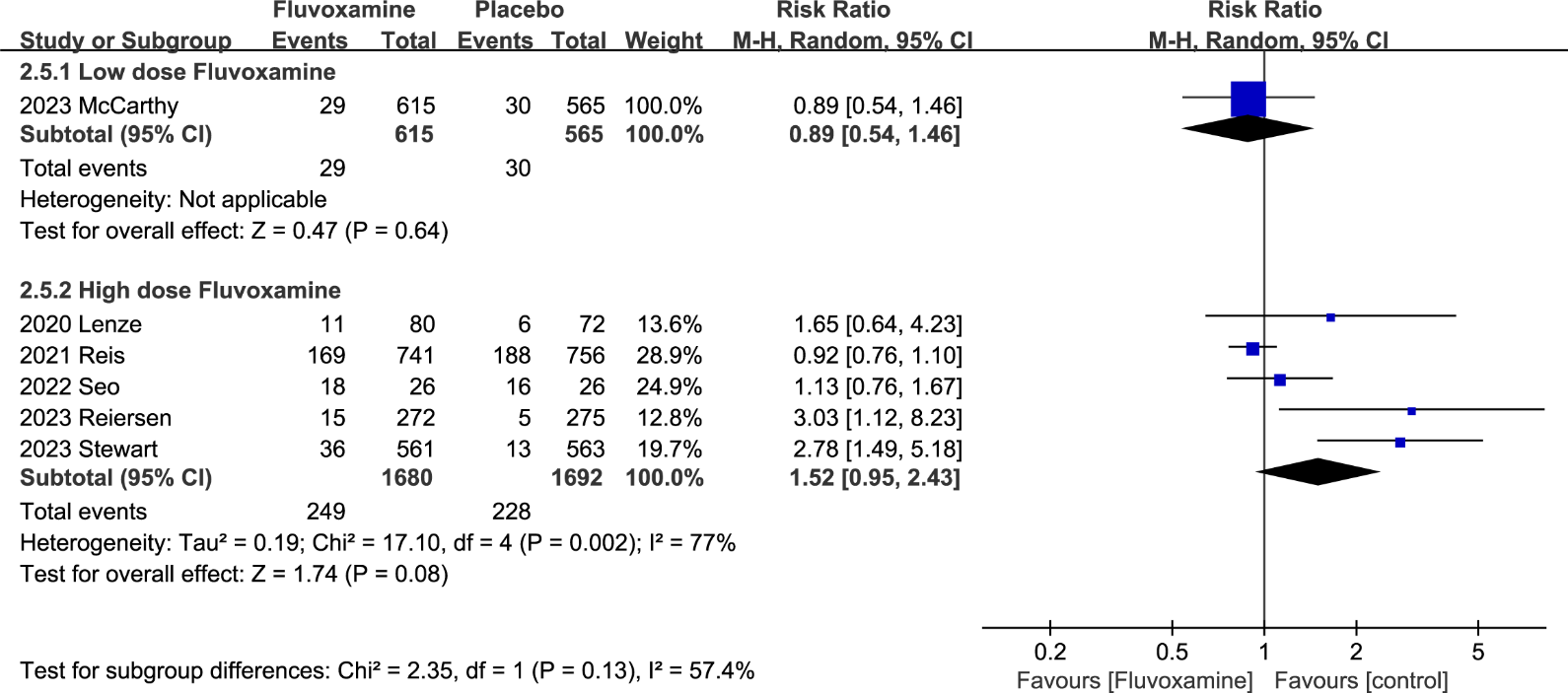

Supplement: S4 Fig — (TIF) [file pone.0300512.s004.tif]

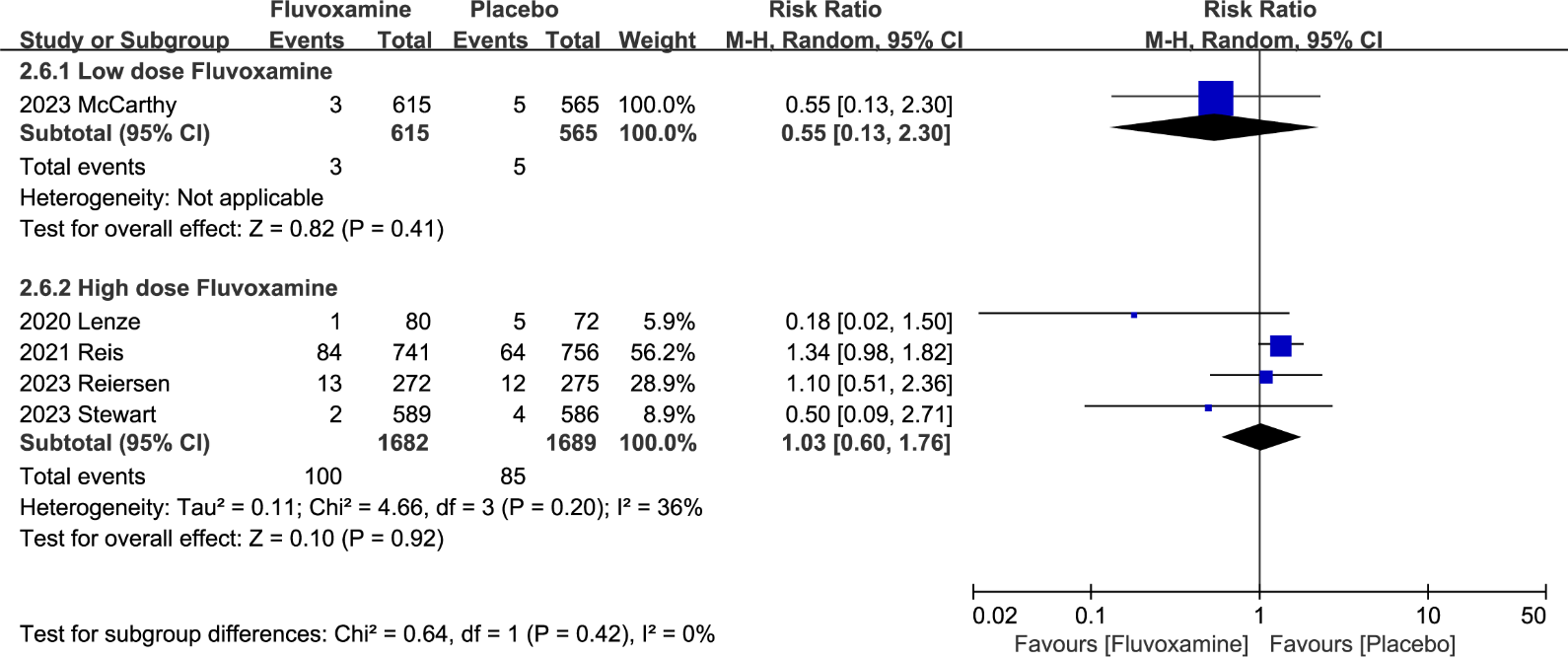

Supplement: S5 Fig — (TIF) [file pone.0300512.s005.tif]
