# Supplementary material for: The efficacy and safety of fluvoxamine in patients with COVID-19: A systematic review and meta-analysis from randomized controlled trials
Source: PLoS One. 2024 May 16;19(5):e0300512. doi: 10.1371/journal.pone.0300512 (PMC11098472; doi:10.1371/journal.pone.0300512)
Supplement: S1 Table — (DOCX) [file pone.0300512.s006.docx]

**Table S1. Detailed search strategy**

**Search Date:** January 31, 2024

**Pubmed：**

| Search | Query | Results |
| --- | --- | --- |
| #1 | (((((((((((((((((((((((((((((((((((COVID 19[Title/Abstract]) OR (2019-nCoV Infection[Title/Abstract])) OR (2019 nCoV Infection[Title/Abstract])) OR (2019-nCoV Infections[Title/Abstract])) OR (Infection, 2019-nCoV[Title/Abstract])) OR (SARS-CoV-2 Infection[Title/Abstract])) OR (Infection, SARS-CoV-2[Title/Abstract])) OR (SARS CoV 2 Infection[Title/Abstract])) OR (SARS-CoV-2 Infections[Title/Abstract])) OR (2019 Novel Coronavirus Disease[Title/Abstract])) OR (2019 Novel Coronavirus Infection[Title/Abstract])) OR (COVID-19 Virus Infection[Title/Abstract])) OR (COVID 19 Virus Infection[Title/Abstract])) OR (COVID-19 Virus Infections[Title/Abstract])) OR (Infection, COVID-19 Virus[Title/Abstract])) OR (Virus Infection, COVID-19[Title/Abstract])) OR (COVID19[Title/Abstract])) OR (Coronavirus Disease 2019[Title/Abstract])) OR (Disease 2019, Coronavirus[Title/Abstract])) OR (Coronavirus Disease-19[Title/Abstract])) OR (Coronavirus Disease 19[Title/Abstract])) OR (Severe Acute Respiratory Syndrome Coronavirus 2 Infection[Title/Abstract])) OR (COVID-19 Virus Disease[Title/Abstract])) OR (COVID 19 Virus Disease[Title/Abstract])) OR (COVID-19 Virus Diseases[Title/Abstract])) OR (Disease, COVID-19 Virus[Title/Abstract])) OR (Virus Disease, COVID-19[Title/Abstract])) OR (SARS Coronavirus 2 Infection[Title/Abstract])) OR (2019-nCoV Disease[Title/Abstract])) OR (2019 nCoV Disease[Title/Abstract])) OR (2019-nCoV Diseases[Title/Abstract])) OR (Disease, 2019-nCoV[Title/Abstract])) OR (COVID-19 Pandemic[Title/Abstract])) OR (COVID 19 Pandemic[Title/Abstract])) OR (Pandemic, COVID-19[Title/Abstract])) OR (COVID-19 Pandemics[Title/Abstract]) | 374,303 |
| #2 | ((((((((((((((((((((((((((((((Fluvoxamine[Title/Abstract]) OR (DU-23000[Title/Abstract])) OR (DU 23000[Title/Abstract])) OR (DU23000[Title/Abstract])) OR (Fluvoxadura[Title/Abstract])) OR (Fluvoxamin AL[Title/Abstract])) OR (Fluvoxamin beta[Title/Abstract])) OR (Fluvoxamin Stada[Title/Abstract])) OR (Fluvoxamin-neuraxpharm[Title/Abstract])) OR (Fluvoxamin neuraxpharm[Title/Abstract])) OR (Fluvoxamin-ratiopharm[Title/Abstract])) OR (Fluvoxamin ratiopharm[Title/Abstract])) OR (ratio-Fluvoxamine[Title/Abstract])) OR (ratio Fluvoxamine[Title/Abstract])) OR (Fluvoxamina Geminis[Title/Abstract])) OR (Geminis, Fluvoxamina[Title/Abstract])) OR (Fluvoxamine Maleate[Title/Abstract])) OR (Fluvoxamine Maleate, (E)-Isomer[Title/Abstract])) OR (Fluvoxamine, (Z)-Isomer[Title/Abstract])) OR (Novo-Fluvoxamine[Title/Abstract])) OR (Novo Fluvoxamine[Title/Abstract])) OR (Nu-Fluvoxamine[Title/Abstract])) OR (Nu Fluvoxamine[Title/Abstract])) OR (PMS-Fluvoxamine[Title/Abstract])) OR (PMS Fluvoxamine[Title/Abstract])) OR (Luvox[Title/Abstract])) OR (Floxyfral[Title/Abstract])) OR (Fevarin[Title/Abstract])) OR (Dumirox[Title/Abstract])) OR (Faverin[Title/Abstract])) OR (Desiflu[Title/Abstract]) | 3,032 |
| #3 | #1 AND #2 | **258** |

**Embass：**

| Search | Query | Results |
| --- | --- | --- |
| #1 | 'coronavirus disease 2019'/exp | 382,515 |
| #2 | '2019 novel coronavirus disease' OR '2019 novel coronavirus epidemic' OR '2019 novel coronavirus infection' OR '2019-ncov disease' OR '2019-ncov infection' OR 'coronavirus disease 2' OR 'coronavirus disease 2010' OR 'coronavirus disease-19' OR 'coronavirus infection 2019' OR 'covid' OR 'covid 19' OR 'covid 2019' OR 'covid-10' OR 'covid-19' OR 'covid19' OR 'ncov 2019 disease' OR 'ncov 2019 infection' OR 'novel coronavirus 2019 disease' OR 'novel coronavirus 2019 infection' OR 'novel coronavirus disease 2019' OR 'novel coronavirus infection 2019' OR 'paucisymptomatic coronavirus disease 2019' OR 'sars coronavirus 2 infection' OR 'sars-cov-2 disease' OR 'sars-cov-2 infection' OR 'sars-cov2 disease' OR 'sars-cov2 infection' OR 'sarscov2 disease' OR 'sarscov2 infection' OR 'severe acute respiratory syndrome 2' OR 'severe acute respiratory syndrome coronavirus 2 infection' OR 'severe acute respiratory syndrome coronavirus 2019 infection' OR 'severe acute respiratory syndrome cov-2 infection' OR 'wuhan coronavirus disease' OR 'wuhan coronavirus infection' OR 'coronavirus disease 2019':ab,ti,kw | 431,798 |
| #3 | #1 OR #2 | 471,235 |
| #4 | 'fluvoxamine'/exp | 14,823 |
| #5 | '5 methoxy 1 [4 (trifluoromethyl) phenyl] 1 pentanone o (2 aminoethyl) oxime' OR '5 methoxy 4` (trifluoromethyl) valerophenone o (2 aminoethyl) oxime' OR 'du 23000' OR 'du23000' OR 'fluoxamine' OR 'fluroxamine' OR 'fluvoxamine':ab,kw,ti | 4,049 |
| #6 | #4 OR #5 | 15,253 |
| #7 | #3 AND #6 | **379** |

**Cochrane:**

| Search | Query | Results |
| --- | --- | --- |
| #1 | MeSH descriptor: [COVID-19] explode all trees | 7441 |
| #2 | MeSH descriptor: [Fluvoxamine] explode all trees | 493 |
| #3 | #1 AND #2 | **26** |

**Clinicaltrials.gov：**

| Search | Query | Results |
| --- | --- | --- |
| #1 | Status:All studies; condition or disease: COVID-19; Intervention: Fluvoxamine; Study type: interventional(Clinical Trial) | **13** |
