# Supplementary material for: The efficacy and safety of fluvoxamine in patients with COVID-19: A systematic review and meta-analysis from randomized controlled trials
Source: PLoS One. 2024 May 16;19(5):e0300512. doi: 10.1371/journal.pone.0300512 (PMC11098472; doi:10.1371/journal.pone.0300512)
Supplement: S2 Table — (DOCX) [file pone.0300512.s007.docx]

**Table S2: Inclusion, exclusion criteria, study design and outcome assessments of the included studies**

| **Trials** | **Lenze et al 2020 (STOP COVID 1)** |
| --- | --- |
| ***Inclusion*** ***Criteria*** | The study included adults living in the community with SARSCoV-2 infection confirmed by polymerase chain reaction assay and who were symptomatic within 7 days of the first dose of study medication. |
| ***Exclusion Criteria*** | Exclusion criteria included having COVID-19 that required hospitalization or evidence of the primary end point with oxygen saturation less than 92% on room air at the time of randomization. Other exclusion criteria were severe underlying lung disease (eg, chronic obstructive pulmonary disease or required home oxygen, interstitial lung disease, pulmonary hypertension), decompensated cirrhosis, congestive heart failure (New York Heart Association class III or IV), or immunocompromised (eg, solid organ transplant recipient or donor, bone marrow transplant recipient, AIDS, or taking immunosuppressant biologic drugs or high dose corticosteroids [>20mg/d of prednisone]. |
| ***Study Design*** | A double-blind, placebo-controlled, randomized clinical trial that compared fluvoxamine with placebo in adult outpatients with confirmed SARS-CoV-2 infection. |
| ***Efficacy Outcomes*** | The primary end point was clinical deterioration defined by both the (1) presence of dyspnea (ie, shortness of breath) or hospitalization for shortness of breath or pneumonia and (2) decrease in oxygen saturation (<92%) on room air or supplemental oxygen requirement to maintain oxygen saturation of 92% or greater. For the secondary end points, episodes of clinical deterioration were rated on a novel 7-point scale with 0 indicating none; 1, shortness of breath and oxygen saturation less than 92% but no supplemental oxygen needed; 2, shortness of breath and oxygen saturation less than 92% plus supplemental oxygen needed; 3, oxygen saturation less than 92% plus supplemental oxygen needed and hospitalization related to dyspnea or hypoxia; 4, oxygen saturation less than 92% plus supplemental oxygen needed and hospitalization related to dyspnea or hypoxia plus ventilator support needed for less than 3 days; 5,oxygen saturation less than 92% plus supplemental oxygen needed and hospitalization related to dyspnea or hypoxia plus ventilator support needed for at least 3 days; and 6, death. The number of days requiring supplemental oxygen, hospitalization, and ventilator support also were assessed. |
| ***Safety Outcomes*** | Adverse events and serious adverse events. |

| **Trials** | **Reis et al 2021 (TOGETHER)** |
| --- | --- |
| ***Inclusion Criteria*** | Patients older than 18 years, presenting to an outpatient care setting with an acute clinical condition consistent with COVID-19 and symptoms beginning within 7 days of the screening date, or positive rapid test for SARS-CoV-2 antigen done at the time of screening or patient with positive SARS-CoV-2 diagnostic test within 7 days of symptom onset. Eligible patients also had at least one additional criterion for high risk: diabetes; systemic arterial hypertension requiring at least one oral medication for treatment; known cardiovascular disease (heart failure, congenital heart disease, valve disease, coronary artery disease, cardiomyopathies being treated, clinically manifested heart disease and with clinical repercussion); symptomatic lung disease or treatment for such (emphysema, fibrosing diseases); symptomatic asthma requiring chronic use of agents to control symptoms; smoking; obesity, defined as body-mass index greater than 30 kg/m² (weight and height information provided by the patient); having had a transplant; stage IV chronic kidney disease or on dialysis; immunosuppression or use of corticosteroid therapy (equivalent to at least 10 mg of prednisone per day) or immunosuppressive therapy; history of cancer in the last 0.5 years or undergoing current cancer treatment or aged 50 years or older; and unvaccinated status. |
| ***Exclusion Criteria*** | Patients who met any of the following key criteria were excluded from the trial: diagnostic examination for SARS-CoV-2 negative associated with acute flu-like symptoms (patients with negative test taken early and becoming positive a few days later were eligible, if they were less than 7 days after the onset of flu-like symptoms); acute respiratory condition compatible with COVID-19 treated in primary care and previously requiring hospitalisation; acute respiratory condition owing to other causes; received vaccination for SARS-CoV-2; dyspnoea secondary to other acute and chronic respiratory causes or infections (eg, decompensated chronic obstructive pulmonary disease, acute bronchitis, pneumonia, primary pulmonary arterial hypertension); current use of SSRIs (use of other serotonin reuptake inhibitors were not excluded); uncontrolled psychiatric disorders or suicidal ideation; inability or unwillingness to follow research guidelines and procedures. |
| ***Study Design*** | A randomised, adaptive platform trial to investigate the efficacy of repurposed treatments for COVID-19 disease among high-risk adult outpatients. |
| ***Efficacy Outcomes*** | Primary outcome was a composite endpoint of medical admission to a hospital setting due to COVID-19-related illness defined as COVID-19 emergency setting visits with participants remaining under observation for more than 6 h or referral to further hospitalisation due to the progression of COVID-19 within 28 days of randomisation. Key secondary outcomes include viral clearance, time to clinical improvement, number of days with respiratory symptoms, time to hospitalisation for any cause or due to COVID-19 progression, all-cause mortality and time to death from any causes, WHO clinical worsening scale score, days in hospital and on ventilator. |
| ***Safety Outcomes*** | Adverse events, adverse reactions to the study medications and serious adverse events. |

| **Trials** | **Seo et al 2022** |
| --- | --- |
| ***Inclusion Criteria*** | This study included adult patients over 18 with SARS-CoV-2 infection laboratory-confirmed by real time-polymerase chain reaction (RT-PCR). Patients who had symptoms consistent with COVID-19 with onset less than 7 days after randomization and had positive RT-PCR results within 3 days of randomization were enrolled. |
| ***Exclusion Criteria*** | Exclusion criteria included patients with severe medical comorbidities such as severe underlying lung disease, chronic liver disease, congestive heart failure (New York Heart Association class III or IV), chronic kidney disease, or those who were immunocompromised. Patients who were referred to a hospital within 24 hours after admission to the CTC with evidence of the primary endpoint at the time of randomization, or those who were referred to a hospital for other reasons without aggravation of COVID-19, were also excluded from the analysis. Foreigners were also excluded because they were unable to provide self-reports written in Korean. |
| ***Study Design*** | A single-blind, randomized, placebo-controlled trial that compared fluvoxamine with placebo in adult patients with confirmed severe acute respiratory syndrome coronavirus 2 (SARS-CoV-2) infection who were admitted to a community treatment center, in Seoul, Korea. |
| ***Efficacy Outcomes*** | The primary endpoint was clinical deterioration defined by any of the following: (1) decrease in oxygen saturation (<94.0%) in room air; (2) supplemental oxygen required in order to maintain an oxygen saturation of 94% or more; (3) aggravation of pneumonia with dyspnea and increased infiltrate on chest X-ray, or minute respiratory rate over 25; (4) WHO clinical progression scale 4 or greater. Secondary endpoints included all the categories of clinical deterioration described above as primary endpoints, and days to clinical deterioration. |
| ***Safety Outcomes*** | Adverse events and serious adverse events. |

| **Trials** | **McCarthy et al 2023 (ACTIV-6 Low)** |
| --- | --- |
| ***Inclusion Criteria*** | The trial sites verified eligibility criteria, including (1) age of 30 years or older, (2) confirmed SARS-CoV-2 infection for 10 days or less, and (3) whether experiencing 2 or more COVID-19 symptoms for 7 days or less from the time of consent. Symptoms included the following: fatigue, dyspnea, fever, cough, nausea, vomiting, diarrhea, body aches, chills, headache, sore throat, nasal symptoms, and loss of sense of taste or smell. |
| ***Exclusion Criteria*** | Exclusion criteria included hospitalization, known allergies or contraindications (including to prohibited concomitant medications), and use of fluvoxamine within 14 days of enrollment. |
| ***Study Design*** | A ongoing double-blind, placebo-controlled platform randomized clinical trial is being conducted using a decentralized approach, allowing for use in a wide range of settings within health care systems and the community. |
| ***Efficacy Outcomes*** | The primary outcome was time to sustained recovery (defined as the third day of 3 consecutive days without symptoms). This was selected a priori from among the 2 coprimary outcomes that remained available to the other drugs. By definition, participants who died did not recover regardless of reported freedom from symptoms. There were 7 secondary outcomes, including hospitalization or death by day 28; the mean amount of time spent feeling unwell; COVID Clinical Progression Scale scores on days 7, 14, and 28; mortality through day 28; and the composite outcome of hospitalization, urgent care visit, emergency department visit, or death through day 28. The final secondary outcome, the PROMIS-29 (Patient-Reported Outcomes Measurement Information System 29) profile, was to be assessed through day 90 and is not reported here because of the longer follow-up. |
| ***Safety Outcomes*** | Adverse events and serious adverse events. |

| **Trials** | **Reiersen et al 2023 (STOP COVID 2)** |
| --- | --- |
| ***Inclusion Criteria*** | Men and woman age 30 and older; not currently hospitalized; proven SARS-CoV-2 positive (per lab or physician report); currently symptomatic with one or more of the following symptoms: fever, cough, myalgia, mild dyspnea, chest pain, diarrhea, nausea, vomiting, anosmia (inability to smell), ageusia (inability to taste), sore throat, nasal congestion; able to provide informed consent; upon initial screening, participant reports one of the following risk factors for clinical deterioration: age≥40, racial/ethnic group African-American, Hispanic, or Native American (including more than one race), or 1+ of the following medical conditions which increase risk for developing moderate-severe COVID illness: obesity, hypertension, diabetes, heart disease (coronary artery disease, history of myocardial infarction, or heart failure), lung disease (eg asthma, COPD), immune disorder (eg rheumatoid arthritis, lupus). |
| ***Exclusion Criteria*** | Illness severe enough to require hospitalization or already meeting study's primary endpoint for clinical worsening; unstable medical comorbidities, per patient report and/or medical records; immunocompromised; taking warfarin, phenytoin, clopidogrel, and St John's wort; taking SSRIs, SNRIs, or tricyclic antidepressants, unless these are at a low dose such that a study investigator concludes that a clinically significant interaction with fluvoxamine; individuals who take alprazolam or diazepam and are unwilling to cut the medication by 25%; participants taking theophylline, tizanidine, clozapine, or olanzapine will be reviewed with a study investigator and excluded unless the investigator concludes that the risk to the participant is low; received vaccine for COVID-19. |
| ***Study Design*** | Participants will be randomly assigned (1:1) to take either fluvoxamine or a placebo. This phase of the study will last approximately 15 days and is double-blinded. Participants will take up to 100mg of fluvoxamine or placebo by mouth twice a day for a daily total of 200mg. Participants will continue this dose for approximately 15 days. Depending on tolerability, the dose may be adjusted. Participants will also complete short 5 minutes assessments daily to report the results of self-monitoring (including oxygen level, blood pressure, and temperature), a shortness of breath rating and any adverse events. |
| ***Efficacy Outcomes*** | Number of participants with clinical deterioration, defined as the number of participants who experienced the following: both of the following: 1) presence of dyspnea and/or hospitalization for shortness of breath or pneumonia, 2) decrease in O2 saturation (<92% on room air) and/or supplemental oxygen requirement to keep O2 saturation ≥92%). |
| ***Safety Outcomes*** | Adverse events and serious adverse events. |

| **Trials** | **Stewart et al 2023 (ACTIV-6 High)** |
| --- | --- |
| ***Inclusion Criteria*** | Study eligibility criteria at the time of screening were confirmed at the site level and included age 30 years or older, SARS-CoV-2 infection confirmed with a positive polymerase chain reaction or antigen test result (including home-based testing) within the past 10 days, and actively experiencing 2 or more COVID-19 symptoms for 7 days or less from the time of consent. |
| ***Exclusion Criteria*** | Individuals meeting the following criteria were excluded from participation: current or recent hospitalization for COVID-19, ongoing or planned participation in other interventional trials for COVID-19, current or recent use (within 14 days) of fluvoxamine or other selective serotonin (or norepinephrine) reuptake inhibitors ormonoamine oxidase inhibitors, bipolar disorder, pregnant or breastfeeding, or known allergy or contraindications to fluvoxamine. |
| ***Study Design*** | ACTIV-6 is a double-blind randomized placebo-controlled platform trial to study repurposed medications for the treatment of outpatients with mild to moderate COVID-19 in the US. |
| ***Efficacy Outcomes*** | The primary outcome was time to sustained recovery within 28 days, defined as the time from receipt of the study drug to the third of 3 consecutive days without COVID-19 symptoms. This measure was selected a priori from the 2 possible primary outcomes of the platform. The other possible primary outcome—time to hospitalization or death—transitioned to a secondary outcome when not selected as the primary outcome, per the statistical analysis plan. Participants who died during follow-up were deemed to have not recovered, regardless of whether they were without symptoms for 3 consecutive days. Secondary outcomes included 3 time-to-event end points administratively censored at day 28: time to death (number of events permitting), time to hospitalization or death (number of events permitting), and time to first health care utilization (a composite of urgent care visits, emergency department visits, hospitalization, or death). Additional secondary outcomes included mean time spent unwell through day 14 and the World Health Organization COVID-19 clinical progression scale score on days 7, 14, and 28. Quality of life measures using the PROMIS-29 (patient reported outcomes measurement information system) are being collected through day 180 and are not included in this report. |
| ***Safety Outcomes*** | Adverse events and serious adverse events. |
